# Editorial: Cognition in Multiple Sclerosis

**DOI:** 10.3389/fneur.2021.751687

**Published:** 2021-08-30

**Authors:** Rosa Cortese, Antonio Carotenuto, Massimiliano Di Filippo, Roberta Lanzillo

**Affiliations:** ^1^Department of Medicine, Surgery and Neuroscience, University of Siena, Siena, Italy; ^2^Department of Neurosciences, Reproductive and Odontostomatological Sciences, Federico II University, Naples, Italy; ^3^Section of Neurology, Department of Medicine and Surgery, University of Perugia, Perugia, Italy

**Keywords:** multiple sclerosis, cognition, cognitive reserve, cognitive rehabilitation, cognitive impairment

Cognitive impairment (CI) is common in patients with multiple sclerosis (MS), with a prevalence ranging from 34 to 65%, depending on several factors, such as disease duration and age at disease onset. Moreover, accumulating evidence reports that in MS less explored cognitive domains (i.e., theory of mind, pragmatics, meta-cognition, prospective memory) might be also affected in the absence of an overall CI (1). Recently, diverse computerized neuropsychological testing tools have been developed to allow the application of more comprehensive assessment batteries. Yet, understanding of their use remains limited due to the lack of validation studies on larger samples. To date, the pathological brain changes associated with cognitive disability in MS are not fully understood (2). The application of novel advanced imaging techniques has the potential to reveal the mechanisms underpinning both the overall CI and the impairment in selected cognitive domains. Finally, efficient approaches for treating CI are still lacking. Despite the efficacy of disease modifying treatment in preventing cognitive decline, results of clinical trials were disappointing (1).

This Research Topic on CI in MS aims to review studies on this subject. Authors have contributed with 15 works on different aspects of MS-related CI, including works exploring (i) CI assessment tools development and the cognitive processes underlying failure at neuropsychological tests in MS; (ii) correlations between CI and disease biomarkers, (iii) MRI pathological substrates underpinning CI; and (iv) possible therapeutic strategies for CI in MS.

In particular, one main field investigated by the authors relates to self-assessment and perception of cognitive functioning. Riccardi et al. developed a new questionnaire called Sclerosis Multipla Autovalutazione Cognitiva (SMAC), which showed a promising Patient-Reported Outcome to be included in MS neuropsychological evaluation. Another way to explore the experience of individuals living with MS and their cognitive involvement is the Cognitive Assessment Interview (CAI), a patient and informant-based semi-structured interview, which Eilam-Stock et al. demonstrated to add information to both self-report measures and neuropsychological assessment, and to characterize the experience of CI in persons living with MS. Interestingly, a common finding of these works is that caregiver perception is more strongly correlated to the objective cognitive performance of people with MS than a patient's self-judgment. It is worth noting, however, that local norms should be followed when interpreting the results of cognitive tests and the performance of regression-based norms developed in other populations need to be considered when applying them to local populations, even when they are from the same country, as demonstrated by Marrie et al..

The main imaging marker of neurodegeneration in MS is supposed to be brain atrophy, which is associated with cognitive impairment and retinal nerve fiber layer (RNFL) atrophy. Fenu et al. confirmed the role of brain atrophy as a biomarker of CI and highlighted the importance of a caregiver's perception for cognitive assessment of patients with MS. In a 5-year follow-up study, Giedraitiene et al. showed that RNFL atrophy and other inflammatory markers, like oligoclonal bands in cerebrospinal fluid were related to cognitive decline in MS patients. Additionally, Portaccio et al. found that Brain Derived Neurotrophic Factor (BDNF) Val66Met polymorphism may have a protective role against cognitive impairment in MS patients.

Diving deeper into the different cognitive functions involved in MS has become a predominant issue. For example, verbal fluency (VF) has been associated with several cognitive functions, but the cognitive processes underlying deficits in this cognitive domain in MS are controversial. Delgado-Álvarez et al. evaluated the cognitive processes related to VF and developed machine-learning algorithms to predict those patients with cognitive deficits using only VF-derived scores. In their work, VF was influenced by many other cognitive processes, mainly including attention-executive functioning, episodic memory, and language. Semantic fluency and clustering were more explained by memory function, while phonemic fluency and switching were more related to executive functioning, supporting that the multiple cognitive components underlying VF tasks in MS could even serve for screening purposes and the detection of executive dysfunction.

Impaired temporal processing of simultaneity/successiveness has been frequently reported in MS, while interval timing has not been investigated in adults nor pediatric MS patients. In pediatric MS patients, Troche et al. suggest that subcortical deficits might underlie typical alterations in speech and visuomotor coordination. However, future studies are needed to confirm these findings.

This special issue also reviews recent advances on MRI techniques and their potential to provide a deeper understanding of the pathological substrates of CI in MS. Zhang et al. summarized recent works assessing the structural and functional connectivity substrates of cognitive impairment in MS, using different diffusion tensor imaging (DTI) measures (e.g., fractional anisotropy, diffusivities) along with tractography-derived white matter (WM), while Amin and Ontaneda review the role of the thalamus in MS. They suggested that thalamic atrophy may represent an ideal biomarker for studies aiming to test neuroprotective strategies or restorative therapies for cognition.

Boscheron et al. explored different patterns of structural and functional connectivity between the hippocampus and the rest of the brain and their possible relevance to memory performances in early MS. They found a differential impairment in memory performance in the early stages of MS and an important interplay between hippocampal-related structural and functional networks and those performances.

Cognitive reserve (CR) could attenuate the impact of the brain burden on cognition in people with MS. Lopez-Soley et al. found that CR has a significant effect on brain structural connectivity in MS patients with severe clinical impairment and it may protect them from cognitive decline regardless of their cognitive status, with brain damage and aging also influencing cognitive performance.

Further knowledge is required regarding the treatment of CI. Hsu et al. studied the effects of transcranial direct current stimulation on cognition, mood, pain, and fatigue in MS through a systematic review and meta-analysis that provided preliminary evidence that transcranial direct current stimulation has a favorable effect on cognitive processing speed, mood disturbance, pain, and fatigue in MS. However, the effects on cognition and fatigue varied based on the specific assessment used.

Interestingly, exercise training was shown to have high potential to beneficially impact cognitive performance in patients with MS. Rademacher et al. demonstrated that high intensity interval training has potentially higher effects on physical fitness and cognition compared to moderate continuous exercise, with a larger beneficial effect in MS patients with impaired cognition than in those with intact cognition. A future randomized controlled trial with cognitive performance as the primary endpoint may confirm the beneficial role of exercise training.

In conclusion, this Research Topic has shown advances in understanding the pathogenic substrates of CI in MS and suggests promising strategies to assess the involvement of different cognitive domains. These findings could contribute to improving the personalized care of CI in people with MS.

## Author Contributions

RC and RL: literature search and drafting the manuscript. AC and MDF: literature search and revising the manuscript. All authors contributed to the article and approved the submitted version.

## Conflict of Interest

The authors declare that the research was conducted in the absence of any commercial or financial relationships that could be construed as a potential conflict of interest.

## Publisher's Note

All claims expressed in this article are solely those of the authors and do not necessarily represent those of their affiliated organizations, or those of the publisher, the editors and the reviewers. Any product that may be evaluated in this article, or claim that may be made by its manufacturer, is not guaranteed or endorsed by the publisher.
